# Zanubrutinib for the treatment of cutaneous relapse of systemic marginal zone lymphoma: A case report

**DOI:** 10.1016/j.jdcr.2025.10.074

**Published:** 2025-11-24

**Authors:** Selena Osman, Lesley Street, Jori Hardin

**Affiliations:** a Division of Dermatology, Department of Medicine, University of Calgary, Calgary, Alberta, Canada; b Division of Hematology, Department of Medicine, University of Calgary, Calgary, Alberta, Canada

**Keywords:** Bruton's tyrosine kinase inhibitor, cutaneous B-cell lymphoma, cutaneous relapse, Marginal zone lymphoma, systemic disease

## Introduction

Marginal zone lymphoma (MZL) is an indolent B-cell non-Hodgkin lymphoma characterized by the proliferation of small B-lymphocytes. Subtypes include extranodal (also known as mucosa-associated lymphoid tissue/MALT lymphoma), splenic, and nodal MZL. In the 2022 International Consensus Classification (ICC), primary cutaneous cases were classified separately as “primary cutaneous marginal zone lymphoproliferative disorders” due to their indolent nature, difference in treatment approach, and favorable prognosis. All other anatomic sites are classified collectively under the term MALT lymphomas - the most common form of MZL.^1^^,^^2^

Zanubrutinib is a highly selective Bruton’s tyrosine kinase (BTK) inhibitor approved for relapsed or refractory MZL. It offers a favorable safety profile and demonstrated significant efficacy in phase II trials.^3^

We describe a patient initially diagnosed with extranodal MZL treated with rituximab and bendamustine, followed by maintenance rituximab with complete response (CR). She subsequently presented with skin-limited relapse 4 years later with excellent response to zanubrutinib. There is limited literature reporting extranodal MZL with subsequent isolated cutaneous relapse and outside of clinical trial data, and there are no reports of zanubrutinib for the treatment of cutaneous MZL.

## Case report

A 78-year-old female presented with 2 plum-colored, indurated nodules on the left arm. Skin biopsy showed a dense dermal lymphoid infiltrate extending into the subcutis, composed of small- to medium-sized lymphocytes with scattered plasma cells. Immunohistochemistry demonstrated neoplastic B-cells positive for CD10 and BCL2 and negative for CD5, BCL6, CD43, TdT, and MUM-1. B-lymphocytes showed lambda light chain restriction. Ki-67 was 3%. Initial staging positron-emission-tomography computed tomography (PET-CT) revealed no hypermetabolic nodes or involvement of the spleen or bone marrow; however, pre-treatment PET-CT showed low-volume hypermetabolic lymph nodes in the axillary, hilar, carinal, inguinal, and retroperitoneal regions with splenomegaly. Baseline investigations demonstrated anemia (hemoglobin of 105 g/L) in addition to an IgM monoclonal protein measuring 27 g/L. Subsequent bone marrow confirmed involvement with indolent B-cell lymphoma, consistent with MZL (negative t(14;18) translocation). Collectively, these findings supported a diagnosis of systemic extranodal MZL with bone marrow and cutaneous involvement.

Rituximab and bendamustine were started with CR on interim CT scan 7 months after initiation. She was subsequently transitioned to maintenance rituximab for 11 months, with a pause during the COVID-19 pandemic. She resumed maintenance rituximab one year later for an additional 10-months.

**Fig 1:**
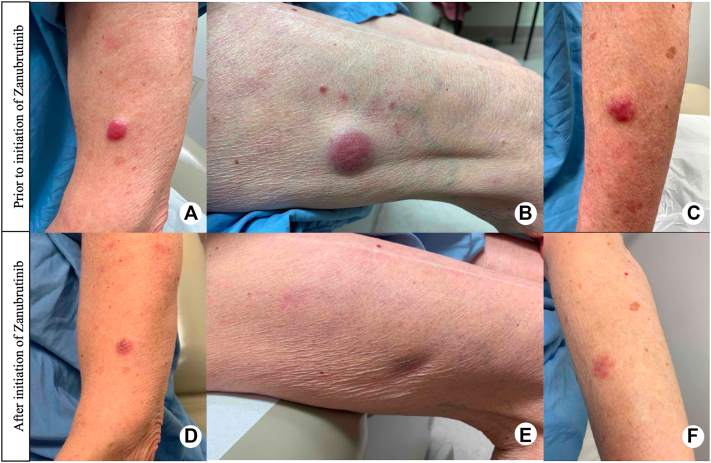
From left to right: **A**) left lateral upper forearm, **B**) right lateral thigh, **C**) left dorsal forearm. Erythematous nodules and tumors prior to the initiation of zanubrutinib (**A**-**C**), with significant improvement 7 months after initiation of zanubrutinib (**D**-**F**). Notably, there was complete resolution at 11-month follow-up.

Four years after her original diagnosis, the patient presented with numerous erythematous, plum-colored papulonodules on her body (Fig 1, *A-C*). A repeat skin biopsy confirmed recurrence of her known MZL. CT of the chest, abdomen, and pelvis did not demonstrate evidence of systemic disease. At the time of skin relapse, hemoglobin was 126 g/L and IgM was 4 g/L, which was diminished compared to the time of completion of chemo-immunotherapy and not felt to represent blood involvement. A diagnosis of skin-limited relapse of MZL was made. The patient continued to develop plum-colored papules, plaques, nodules, and tumors on the arms, thighs, breast, trunk, and back—therefore, zanubrutinib 160 mg orally twice daily was started. Within a few weeks, there was regression in the size of lesions and at 11-month follow-up, she had CR to treatment with no adverse effects. The patient remains on zanubrutinib with no recurrence of disease (Fig 1, *D-F*).

## Discussion

MZLs are a heterogenous group of indolent non-Hodgkin lymphomas that arise from memory B-lymphocytes. Extranodal MZL is the best characterized and most common subtype with the best prognosis (5-year relative survival rate of 94%).^4^ Extranodal MZL can manifest in any tissue, but commonly affects the stomach, eye/adnexa, skin, lung, and parotid gland. The neoplastic B-cells of MALT lymphoma are small with mature chromatin and round to slightly irregular nuclear contours, with variable amounts of cytoplasm.^1^ Immunohistochemistry is generally negative for CD5, CD10, CD23, LEF1, Cyclin D1, and SOX11, though rare CD5 or CD10 positive cases have been described.^1^ Flow cytometric analysis or in situ hybridization can be used to identify light chain restriction along with IgH rearrangements and negative t(14;18).^1^ Most MALT lymphomas remain localized, though multifocal organ involvement or disseminated disease can occur in up to 25% of cases.^1^^,^^4^

We report a patient with extranodal MZL with multifocal organ involvement who had CR to anti-CD20 therapy and bendamustine, however, later presented with skin-limited relapse. She demonstrated CR to targeted therapy with zanubrutinib.

Zanubrutinib is an irreversible inhibitor of BTK that was designed to be more selective than other BTK inhibitors with improved oral absorption. There have been 2 clinical trials which led to the accelerated approval of zanubrutinib for relapsed/refractory (R/R) MZL. The first was a phase 1/2 multicenter study which included 20 patients with R/R MZL, with extranodal disease,^5^ with nodal, and 6 with splenic MZL subtypes.^6^ Most patients had received anti-CD20 therapy with the median prior lines of therapy being 2. The overall response rate (ORR) was 80% with complete remission (CR) in 20% of patients, and the best ORR was 100% in the nodal MZL group, compared to 88.9% and 50% in the extranodal and splenic MZL groups, respectively.^6^ The second clinical trial was the phase 2 MAGNOLIA trial, which included 68 patients with R/R MZL who had been previously treated with an anti-CD20 agent.^7^ 77.9% of patients had extranodal disease at baseline affecting non-gastric and non-cutaneous sites in the majority (73.1%), followed by skin involvement in 15.4%, and gastric involvement in 7.7%. The reported ORR was 68.2% and was similar among all MZL subtypes, with CR in 25.8% of patients.^7^ The final analysis of the trial with median follow-up of 28 months, and median duration of treatment of 2 months, showed that the ORR was 64%, 76%, 66.7%, and 50% in extranodal, nodal, splenic, and unknown subtypes (concurrent nodal and extranodal disease), respectively. Progression free survival was 71%.^8^ There was no further stratification in either trial of ORR by extranodal site involvement to elucidate the efficacy of zanubrutinib in cutaneous disease.

We report the case of a 78-year-old female initially diagnosed with multiorgan extranodal MZL treated with rituximab and bendamustine who later developed skin-limited relapse with CR to zanubrutinib. Zanubrutinib is a well-tolerated, chemotherapy-free approach to managing skin-limited relapse of systemic MZL.

## Conflicts of interest

None disclosed.
